# Septic arthritis of the knee due to *Pantoea* *agglomerans*: look for the thorn

**DOI:** 10.5194/jbji-6-51-2020

**Published:** 2020-12-15

**Authors:** Tobias Koester, Taro Kusano, Henk Eijer, Robert Escher, Gabriel Waldegg

**Affiliations:** 1 Department of Orthopedics and Traumatology, Emmental Hospital, Burgdorf, Switzerland; 2 Department of Internal Medicine and Infectious Diseases, Emmental Hospital, Burgdorf, Switzerland

## Abstract

We report on a patient with septic arthritis of the knee with *Pantoea agglomerans* after a penetrating black locust thorn injury. Antibiotics alone or in combination with an arthroscopy may be insufficient for achieving source control. Accurate medical history and open debridement with a search for a thorn fragment are key to successful treatment.

## 1 Introduction

*Pantoea agglomerans* is a facultative anaerobe and environmental yellow-pigmented bacterium of the family Enterobacteriaceae found on plants, in the earth and water, and occasionally in wounds of animals. The bacterium may cause human pathogen opportunistic infections and is sometimes responsible for septic arthritis after an injury with a populated thorn.

We describe a patient with septic arthritis of his knee with a human pathogenic strain of *P. agglomerans* after a black locust thorn injury and performed a thorough review of the literature describing this entity caused by the bacterium.

**Figure 1:**
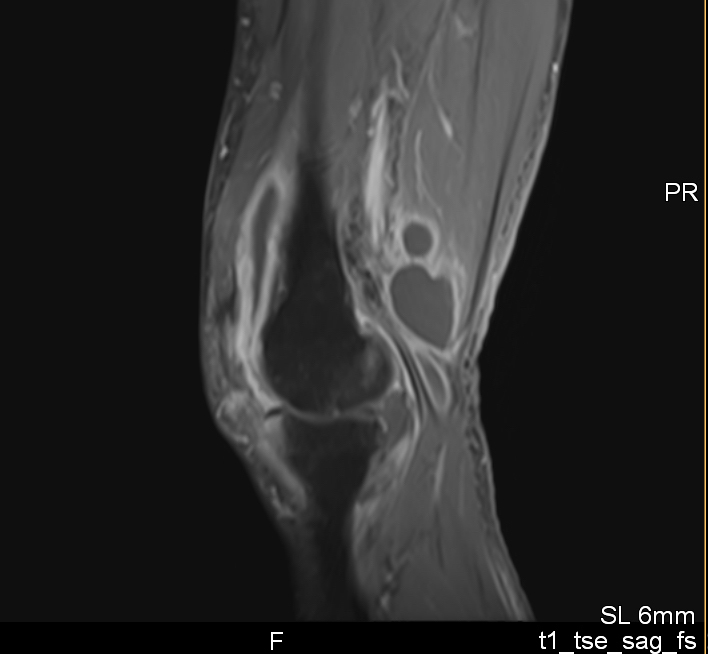
Sagittal MRI slice (T1 fat suppression) of the left knee showed massive synovitis where the thorn fragment was located.

**Figure 2:**
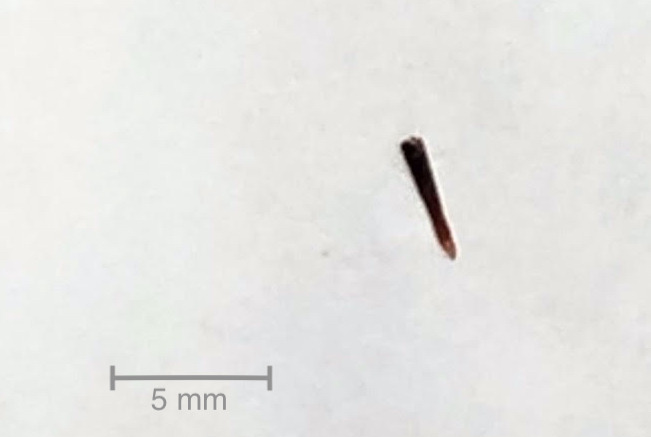
Thorn fragment (4.5 mm) removed during arthrotomy from the suprapatellar pouch.

## 2 Case

In October 2018, a 55-year-old healthy male presented to our emergency department with a swollen and painful left knee. Fifteen days before he had struck his knee against a wooden branch and described symptoms consistent with a distortion. He was afebrile, in good general health, and had normal vital parameters on initial assessment. His knee was swollen, and flexion and extension of the knee were limited due to the swelling. A pinprick-sized superficial skin lesion was located on his thigh just above the knee. Initial X-ray at the emergency department showed intra-articular effusion. His peripheral blood leucocyte count was $10.9\times 10^{9}$ $L^{-1}$ (3.9–$10.2\times 10^{9}$ $L^{-1}$) and the C-reactive protein (CRP) was 100 $\mathrm{mg}\;L^{-1}$ (<5 $\mathrm{mg}\;L^{-1}$). An MRI confirmed severe effusion, generalised tibial bone marrow edema, signs of a partial rupture of the anterior cruciate ligament, and a lesion of the medial meniscus. The radiological findings were interpreted as a possible result of the trauma. Nevertheless, because of the inconclusive elevated inflammatory parameters, we decided to perform an arthrocentesis. The aspirate showed a voluminous non-bloody cloudy fluid with a leukocyte count of 65 000 $µL^{-1}$, 95 % of them polymorphonuclear, and no evidence of crystals. Microscopy with Gram staining was negative, and there was no growth of bacteria following culture of the aspirate within the first days. The patient reported an improvement in pain after aspiration, and flexion and extension improved to the extent that the patient was able to walk without crutches. So, the initial swelling and elevated CRP were attributed to the traumatic partial rupture of the anterior cruciate ligament. Treatment was initiated with analgesics and physiotherapy. However, after prolonged incubation of the joint fluid in liquid media, *P. agglomerans* was isolated. Susceptibility testing according to CLSI standards revealed resistance to aminopenicillin and cephalotin. Initially, the etiologic significance of the bacterium and pathogenicity was uncertain. The patient was recalled for repeated examination, which showed again increased swelling and tenderness. Blood leucocyte count was now $11.9\times 10^{9}$ $L^{-1}$ and the CRP was 43 $\mathrm{mg}\;L^{-1}$. Due to intensified anamnesis the patient also remembered the wooden branch to have had thorns and thought that the scratch could have been caused by a thorn. Based on the positive cultures, an arthroscopy of the knee was performed and intravenous antibiotic treatment with ceftriaxone 2 g daily was started. Cultures of the probes taken during the arthroscopy yielded the same *P. agglomerans*. Therefore, a second-look arthroscopy was performed, which demonstrated an isolated synovitis and intact cruciate ligament. Despite once daily intravenous ceftriaxone for more than 2 weeks, there were no signs of clinical improvement in the follow-up period. A second MRI showed persistent massive synovitis and accumulation of fluid, especially posteriorly (Fig. 1). Because of a high suspicion of a remaining foreign body contributing to persistence of infection, we decided to perform an arthrotomy with a radical synovectomy through a dorsal and ventral approach. Posteriorly we found purulent fluid inside the Baker's cyst, which we excised. A foreign body was not visualised. After completing the dorsal approach and opening the front of the knee, a 4.5 mm thorn fragment was found in the area of synovitis within the suprapatellar pouch (Fig. 2). This explained the failed course of treatment and prolonged course of the infection. Following the intraoperative removal of the foreign body, the clinical condition of the patient steadily improved, with leucocyte count and CRP slowly normalising over the course of 2 weeks.

Antibiotic treatment was continued with intravenous ceftriaxone 2 g once daily, followed by oral ciprofloxacin 500 mg twice daily for a total duration of 4 weeks. The patient regained full range of motion and had almost no pain on follow-up at 5 months.

**Figure 3:**
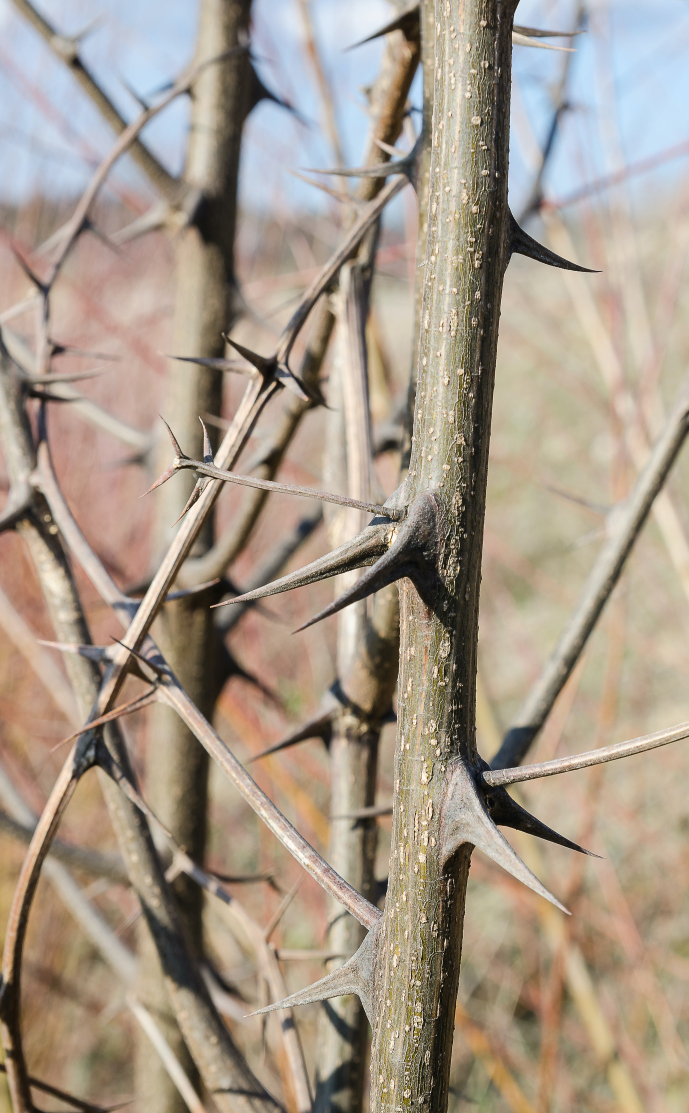
*Robinia pseudoacacia*, commonly known as black locust, populated with *Pantoea agglomerans.* Picture from place of accident near Zurich, Switzerland.

Given our interest and the interest of the patient as to the cause of his infection, he returned to the place of his accident and took thorns of the local plants. The thorns belonged to a plant known as *Robinia pseudoacacia*, a widespread neophytic hardwood tree, commonly known as black locust (Fig. 3). These were cultivated and the isolated *P. agglomerans* showed the identical wild-type susceptibility pattern correlating with the source of the infection.

**Table 1:** Case reports with septic arthritis or synovitis of the joint due to an infection with *Pantoea agglomerans*.

Author and year of	Time to	Age	Source of foreign	Site of	Operative intervention	Foreign body
publication	consultation		body	penetration		retrieved
Flatauer and Khan (1978)	5 d	11	Wooden splinter	Knee	None	–
Stromqvist et al. (1985)	Same day	7	Blackthorn bush	Wrist	Arthroscopy, following arthrotomy	Yes
Vincent and Szabo (1988)	2 weeks	8	Rose thorn	Wrist	Arthrotomy	Yes
Olenginski et al. (1991)	5 d	13	Pricker bush	Hand	Open exploration	Yes
De Champs et al. (2000)	3 weeks	13	Bush thorn	Knee	Arthroscopy	No
De Champs et al. (2000)	Not stated	36	Wooden fence	Hand	Arthroscopy	No
Kratz et al. (2003)	6 weeks	14	Palm tree thorn	Knee	2× arthrotomy	Yes
Ulloa-Gutierrez et al. (2004)	3 d	9	Lemon tree thorn	Knee	Arthrotomy	Yes
Cruz et al. (2007)	5 weeks	8	Unkown	Knee	None	–
Duerinckx et al. (2008)	2 weeks	56	Rosacea thorn	Knee	Arthroscopy	No
Jain et al. (2012)	4 weeks	25	Unknown	Thigh	Open exploration	No
Rave et al. (2012)	Not stated	4	Unkown	Knee	Arthroscopy	Yes
Sharma et al. (2012)	13 weeks	40	Unknown	Knee	None	–
Fianyo et al. (2015)	10 weeks	58	Palm tree thorn	Wrist	Arthroscopy	No
Fianyo et al. (2015)	10 weeks	58	Palm tree thorn	Wrist	Arthroscopy	No
Demicran et al. (2020)	2 weeks	7	Unknown	Knee	Arthrotomy	Yes
Our patient	15 d	55	*Robinia pseudoacacia*	Knee	2× arthroscopy, following arthrotomy	Yes

## 3 Literature review

We performed a literature review in PubMed and Google Scholar for all cases with septic arthritis or synovitis due to *P. agglomerans* from the first case described in 1978 until 2020. We found 16 papers fitting these criteria (Table 1). The age of the patients varied from 4 to 58 years with a median of 13.5 years. The median time to presentation after contact with a plant or potential injury was 2 weeks, with a range from 1 d to 13 weeks. Five cases in Europe have been described, and with no previously reported cases in Switzerland.

A broad variety of thorn plants associated with septic arthritis have been described. Frequent examples include the palm thorn (Sugarman et al., 1977; Kratz et al., 2003; Fianyo et al., 2015), date palm (Ramanathan and Luiz, 1990), and blackthorn bush (Kelly, 1966; Blake et al., 1981; Vincent and Szabo, 1988). Others include rose, cactus, bougainvillea, rosacea, lemon tree, or agave (Simmons et al., 2017; Vincent and Szabo, 1988; Ulloa-Gutierrez et al., 2004; Duerinckx, 2008). Palm thorn and date palm thorn are typically found in central and South America and the Middle East, while blackthorn bush is native to Europe and western Asia.

Thorn injuries may be associated with differing kinds of cutaneous microorganisms, most commonly *Staphylococcus* species or environmental microorganisms like *Nocardia* or *P. agglomerans* (Dutkiewicz et al., 2016).

*P. agglomerans*, formerly called *Enterobacter agglomerans*, is a ubiquitous Gram-negative, slow-growing bacterium. It is naturally found in a variety of different environmental habitats and primarily known as a cause of various plant diseases (Dutkiewicz et al., 2016; Flatauer and Khan, 1978). The bacterium has also been investigated for its relevance in agriculture. In the United States certain strains of *Pantoea* are used as microbial pesticides. In the European Union the species is classified as a level-2 biosafety hazard and the use of all strains as biocontrol agents is prohibited (Rezzonico et al., 2009).

It is not known exactly which strains of the species are pathogenic for humans (Dutkiewicz et al., 2016; Rezzonico et al., 2009). A variety of strains can cause localised wound infections or more systemic infections, especially in immunosuppressed patients (Dutkiewicz et al., 2016). Other less frequently observed manifestations are peritonitis, liver abscess, periodontal disease, pneumonia, prosthetic joint infection, septic arthritis, or osteomyelitis (Cruz et al., 2007).

Studies of thorn injures in general showed an average of 4.2 weeks from injury to first medical consultation, with a time lag of up to 9 months until correct diagnosis of a thorn-associated septic arthritis (Kratz et al., 2003; Kelly, 1966; Baskar et al., 2006). Delay in diagnosis and initiation of treatment is common in thorn-associated arthritis (Simmons et al., 2017). A small injury or scratch from a thorn may easily be forgotten or not be noticed at all, which may account for further postponement in definitive management. Specific questioning about the possibility of a thorn injury is necessary to clarify the sequence of the trauma. This highlights the importance of accurate medical history. Premature categorisation and closure of the case are dangerous and should be avoided.

The diagnosis of an intra-articular thorn fragment is challenging as thorns are mostly radiolucent and cannot be seen on radiographs (Tung et al., 2007). Ultrasound may be used but is an insensitive test, as thorn fragments may be small, their position may not be known, and they may migrate from the original site of penetration. MRI is the most sensitive diagnostic tool (Said et al., 2011; Jain et al., 2012), but a thorn fragment may still be obscured by synovitis.

The fact that *P. agglomerans* is slow-growing may make the microbiological diagnosis difficult (Dutkiewicz et al., 2016; Cruz et al., 2007). In 13 out of 16 of the reviewed papers, antibiotic therapy alone was not successful (Table 1). An arthroscopy or even an open debridement in septic arthritis is necessary. Frequently, aminopenicillins in combination with β-lactamase inhibitors and first- or second-generation cephalosporins are used for suspected Gram-positive cocci. *P. agglomerans* and other Enterobacteriaceae may produce β-lactamase and are not always covered by the conventional empirical antibiotic treatment schemes for septic arthritis.

Thorn fragments were found in 7 out of 13 patients where surgery was performed. Arthroscopic removal of the thorn fragment was attempted in seven cases but was only successful once, while open exploration or arthrotomy was successful in six out of seven patients (Olenginski et al., 1991; De Champs et al., 2000; Kratz et al., 2003; Stromqvist et al., 1985; Rave et al., 2012; Demircan et al., 2020; Vincent and Szabo, 1988). Arthroscopy may be reasonable as an initial strategy to locate a thorn, thus obviating open exploration, but this strategy has not been successful in the majority of described cases. In cases with high suspicion for remaining thorns, open exploration is the best choice for early source control.

Our experience demonstrates again how difficult it can be to find the definitive cause of infection. Similarly to most case reports, our patient presented a prolonged delay after injury, an accurate medical history was not obtained, and the patient did not respond to initial antibiotic treatment. The initial diagnostic strategy proved to be inadequate in detecting a foreign body. Similarly to almost all arthroscopic attempts to control the infection and eradicate possible remaining foreign bodies, we were unsuccessful twice. Without the decision for open exploration with an extensive search for a foreign body, source control of infection would not have been achieved, with the consequence of continued destruction of the joint.

## 4 Conclusion

A precise medical history and recognising *P. agglomerans* as a plant-associated bacterium causing infections by penetrating trauma are key to establishing the correct diagnosis, even if there are difficulties in demonstrating the presence of a thorn with MRI or ultrasound. If source control cannot be achieved and antibiotic treatment fails, the attending physician should clarify the medical history and keep a high suspicion for an occult foreign body. Early open debridement of the joint and an extensive search for the thorn or parts of it seem justified when culture of synovial fluid yields *P. agglomerans*.

## Ethical statement

In accordance with the guidelines of the Ethics Committee of Bern, Switzerland, we obtained signed informed consent of the patient for the purpose of publishing a case report.

## Data Availability

No data sets were used in this article.
